# Even a Chronic Mild Hyperglycemia Affects Membrane Fluidity and Lipoperoxidation in Placental Mitochondria in Wistar Rats

**DOI:** 10.1371/journal.pone.0143778

**Published:** 2015-12-02

**Authors:** María del Consuelo Figueroa-García, María Teresa Espinosa-García, Federico Martinez-Montes, Martín Palomar-Morales, Ricardo Mejía-Zepeda

**Affiliations:** 1 Departamento de Toxicología, Facultad de Medicina Veterinaria y Zootecnia, UNAM, Mexico City, Mexico; 2 Departamento de Bioquímica, Facultad de Medicina, UNAM, Mexico City, Mexico; 3 Unidad de Morfología y Función, Facultad de Estudios Superiores Iztacala, UNAM, Tlalnepantla, State of Mexico, Mexico; 4 Unidad de Biomedicina, Facultad de Estudios Superiores Iztacala, UNAM, Tlalnepantla, State of Mexico, Mexico; Institute for Health & the Environment, UNITED STATES

## Abstract

It is known the deleterious effects of diabetes on embryos, but the effects of diabetes on placenta and its mitochondria are still not well known. In this work we generated a mild hyperglycemia model in female wistar rats by intraperitoneal injection of streptozotocin in 48 hours-old rats. The sexual maturity onset of the female rats was delayed around 6–7 weeks and at 16 weeks-old they were mated, and sacrificed at day 19th of pregnancy. In placental total tissue and isolated mitochondria, the fatty acids composition was analyzed by gas chromatography, and lipoperoxidation was measured by thiobarbituric acid reactive substances. Membrane fluidity in mitochondria was measured with the excimer forming probe dipyrenylpropane and mitochondrial function was measured with a Clark-type electrode. The results show that even a chronic mild hyperglycemia increases lipoperoxidation and decreases mitochondrial function in placenta. Simultaneously, placental fatty acids metabolism in total tissue is modified but in a different way than in placental mitochondria. Whereas the chronic mild hyperglycemia induced a decrease in unsaturated to saturated fatty acids ratio (U/S) in placental total tissue, the ratio increased in placental mitochondria. The measurements of membrane fluidity showed that fluidity of placenta mitochondrial membranes increased with hyperglycemia, showing consistency with the fatty acids composition through the U/S index. The thermotropic characteristics of mitochondrial membranes were changed, showing lower transition temperature and activation energies. All of these data together demonstrate that even a chronic mild hyperglycemia during pregnancy of early reproductive Wistar rats, generates an increment of lipoperoxidation, an increase of placental mitochondrial membrane fluidity apparently derived from changes in fatty acids composition and consequently, mitochondrial malfunction.

## Introduction

The placenta is an autonomous and transient organ of embryonic origin, essential for feeding and oxygenation of the fetus during intrauterine life. Neonatal complications and intrauterine death have been associated with placental insufficiencies that are more common in diabetic pregnancies [1]. In mothers with diabetes mellitus (DM), the *in utero* environment is modified by the hormonal and metabolic alterations and consequently there is an abnormal pattern of fetal growth [2]. Once the fetal development is compromised, there are severe metabolic consequences, including higher risk to develop glucose intolerance, obesity, and diabetes in adolescence and adulthood [3,4]. DM affects the placenta inducing structural and functional changes, and their extent depends of several variables, i.e. the quality of the glycemic control during critical periods in placental development, along with the modality of the treatments, and the events of departures from an appropriate metabolic control of a non-diabetic environment [5]. Due to the increased placental mitochondrial activity and production of reactive oxygen species (ROS), the pregnancy is a state unavoidably associated with oxidative stress. It is well known that under conditions of elevated metabolism or increased mitochondrial activity, many tissue specific cells are more often subject to insult from ROS [6]. Overproduction of ROS may take place at certain stages in placental development and in pathologic pregnancies, such as those complicated by preeclampsia and/or diabetes, overpowering the antioxidant defenses with deleterious results [7]. The mitochondria are dynamic organelles frequently changing shape and distribution [8] and, in order to maintain correct mitochondrial morphology and function, the lipid composition is critical [9]. Physicochemical characteristics such as the membrane fluidity depends largely of lipid composition [10] in such a way that changes in mitochondrial lipid composition might drive to suboptimal mitochondrial function, especially in integral proteins. In mitochondria from the hearts of rats induced to Type 1 Diabetes, a negative association between membrane fluidity and membrane potential has been reported [11], although Type 1 Diabetes usually implies extreme exposure to hyperglycemia. The damage inflicted by ROS has been implicated in DM pathogenesis and its complications [6,12,13]. The mitochondrial function is reflected in its structure and morphology [14–17] but maybe also in its membranal thermotropic characteristics. The aim of this work was to demonstrate that even a chronic mild hyperglycemia is capable of affecting mitochondrial activity and lipoperoxides production during pregnancy, as well as the mitochondrial membrane fluidity and the fatty acids composition of rats at early sexual maturity.

## Material and Methods

### Chemicals

n-Hexane HPLC grade and all other analytical solvents, were purchased from J.T. Baker. Individual and mixtures of fatty acids methyl ester standards, boron trifluoride 14% in methanol, streptozotocin, and several other analytical reagents, were obtained from Sigma Chemical, Co. 1,3-di(1-pyrenyl)propane (DPyP) was obtained from Molecular Probes (Eugene, OR, USA). Purified double distilled water was used for analytical procedures.

### Experimental animals

Wistar rats were maintained under controlled conditions (12-h light/12-h dark cycle, 22°C, and 25% humidity) with free access to water and Harlan rat chow 2018. The experimental protocol was approved by the Institutional Committee for Use and Care of Animals of Experimentation (CICUAE in spanish) of the Facultad de Medicina Veterinaria y Zootecnia—Universidad Nacional Autónoma de México. The guidelines of the institutional bioethics committee and the Federal Regulations for Animal Experimentation and Care (NOM 062-Z00-1999, Ministry of Agriculture, Mexico) were followed. Diabetes was induced in two-days-old female rats by a single intraperitoneal injection of streptozotocin (STZ), at 135 mg/kg of body weight, dissolved in 50 μl of 100 mM citrate buffer, pH 4.5. Control rats were injected with citrate buffer alone. The 60% of the induced rats survived. Five days after birth, neonates exhibiting glycemia greater than 11 mmol/L were considered diabetic. The nursery of rats lasted 4 weeks and 10 control and 10 hyperglycemic rats were selected for the experiments. The condition of the animals was monitored daily. The spontaneous evolution of this treatment led to neonatal STZ-induced diabetic state in the adult as described previously [18]. The onset of sexual maturity was monitored by estrous cycle onset since the eight week; vaginal cells were washed off with PBS from female rats, cells were dyed with toluidine blue 0.1% and analyzed at the microscope. The presence of nucleated cells indicates the beginning of sexual maturity. Blood glucose was determined weekly and measured at noon every time by the glucose-oxidase method. At 16 weeks-old, hyperglycemic and control female rats were mated overnight with normal males. When sperm were found in the vaginal smear on the following day, it was designated as day 0.5 of pregnancy. On day 19 of pregnancy, animals were anesthetized with sodium pentobarbital (40 mg/kg of body weight) for obtaining the organs and sacrificed by exsanguination during the anesthesia. Placenta and liver were removed, washed with PBS and used for analytical procedures. Morphometric parameters of placenta and fetuses were registered.

### Isolation of mitochondria and measurement of lipoperoxidation

Liver and placental mitochondria were isolated by differential centrifugation from tissue homogenates obtained from adult female Wistar rats on day 19 of pregnancy, following the method previously described [19]; protein was determined by Bradford [20] and used for calculations of mitochondrial activity and lipid peroxidation. Lipid peroxidation in mitochondria and total tissue (placenta or liver), with the formation of malondialdehyde (MDA), was assessed by the thiobarbituric acid reactive substances (TBARS) assay [21].

### Mitochondrial respiratory activities and respiratory control

The O2 uptake was determined with a Clark-type electrode in an air-saturated (0.24 mM O2) reaction medium consisting of 250 mmol/L sucrose, 4 mmol/L Tris-HCl, 10 mmol/L MgCl2, 1 mmol/L H3PO4, and 1 mmol/L EGTA, 0.1% BSA, pH 7.4 (respiration medium) and containing 0.5 mg of mitochondrial protein/ml. O2 uptake was determined with 100 μmol/L succinate as substrate, in the presence (state 3) and absence (state 4) of the phosphate acceptor (0.2 mmol/L ADP). O2 uptake was expressed in nAt O2/min/mg protein.

### Analysis of fatty acids composition

Total lipids from placenta were extracted by the method of Folch [22]; total lipids from mitochondrial fraction were extracted as previously described [23] for ghost erythrocytes. Lipids were transesterified with boron trifluoride 14% in methanol [24]. Fatty acids methyl ester composition was determined in a gas chromatograph Clarus 500 of Perkin Elmer controlled by computer. The fatty acids composition is reported as mol%. The fluidity index (U/S) was obtained from the fatty acids composition dividing total unsaturated by total saturated fatty acids.

### Mitochondrial membrane fluidity

Fluorescent labeling of mitochondrial membranes with dipyrenylpropane (DPyP) was performed by adding the appropriate volume of 89 μmol/L DPyP in ethanol into 500 μl of membrane suspension in phosphate buffer, and sonicated in an ultrasonic water bath at room temperature for 1 min. The membranes were incubated in cold room under continuous magnetic stirring for 24 h. Then, the membranes were diluted with phosphate buffer to 3 ml final volume and sonicated 1 min as formerly. The final DPyP concentration was 0.15 μmol/L and membrane phospholipids higher than 0.375 mmol/L, such that the molar ratio of fluorophore to phospholipids was over 1:2500. Fluorescence of DPyP incorporated into membranes was measured in a computer-controlled Perkin-Elmer LS55 fluorescence spectrometer connected to a circulating water bath. The fluorophore was excited at 337 nm and the monomer and excimer fluorescence intensities were read at 385 and 480 nm, respectively. Fluorescence intensities were recorded at several temperatures from 10 to 60°C, equilibrating at least for 5 min before measurements. Light scattering corrections were applied to all fluorescence values from membranes without DPyP.

### Statistical analysis

All data are expressed as mean ± SD. Differences between the groups were compared using statistical analysis performed using JMP 7 software for T Student test (*p* < 0.05) for sexual maturity, blood glucose and physiological parameters and membrane fluidity analysis and its thermotropic parameters of mitochondrial membranes, where one-way ANOVA followed by Tukey test, was applied.

## Results

### Onset of sexual maturity, blood glucose and physiological parameters

**Fig 1:**
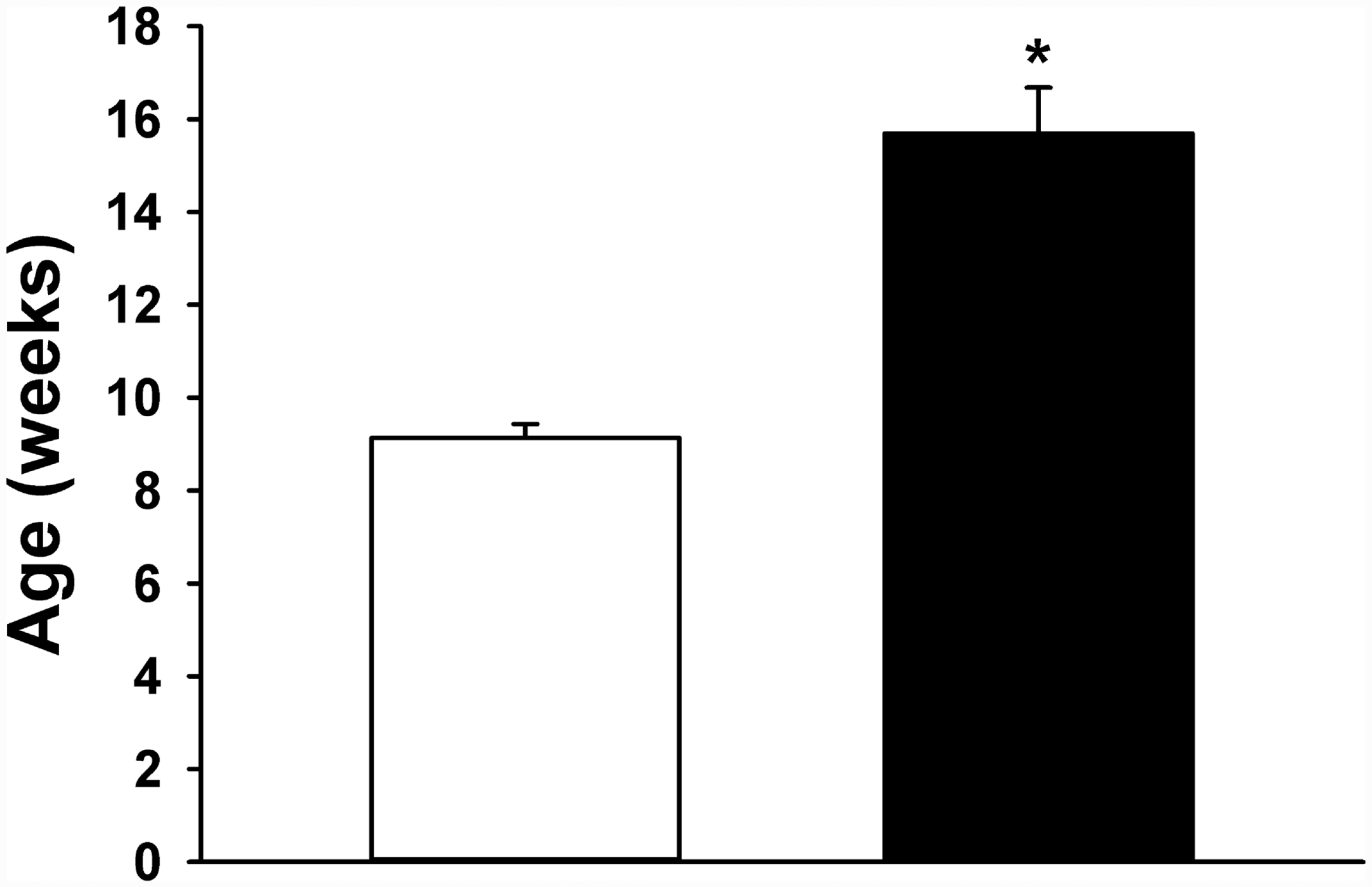
Time for rats to reach sexual maturity. Control rats (white bar) and induced to hyperglycemia (black bar). n = 10. * *p* < 0.05.

**Fig 2:**
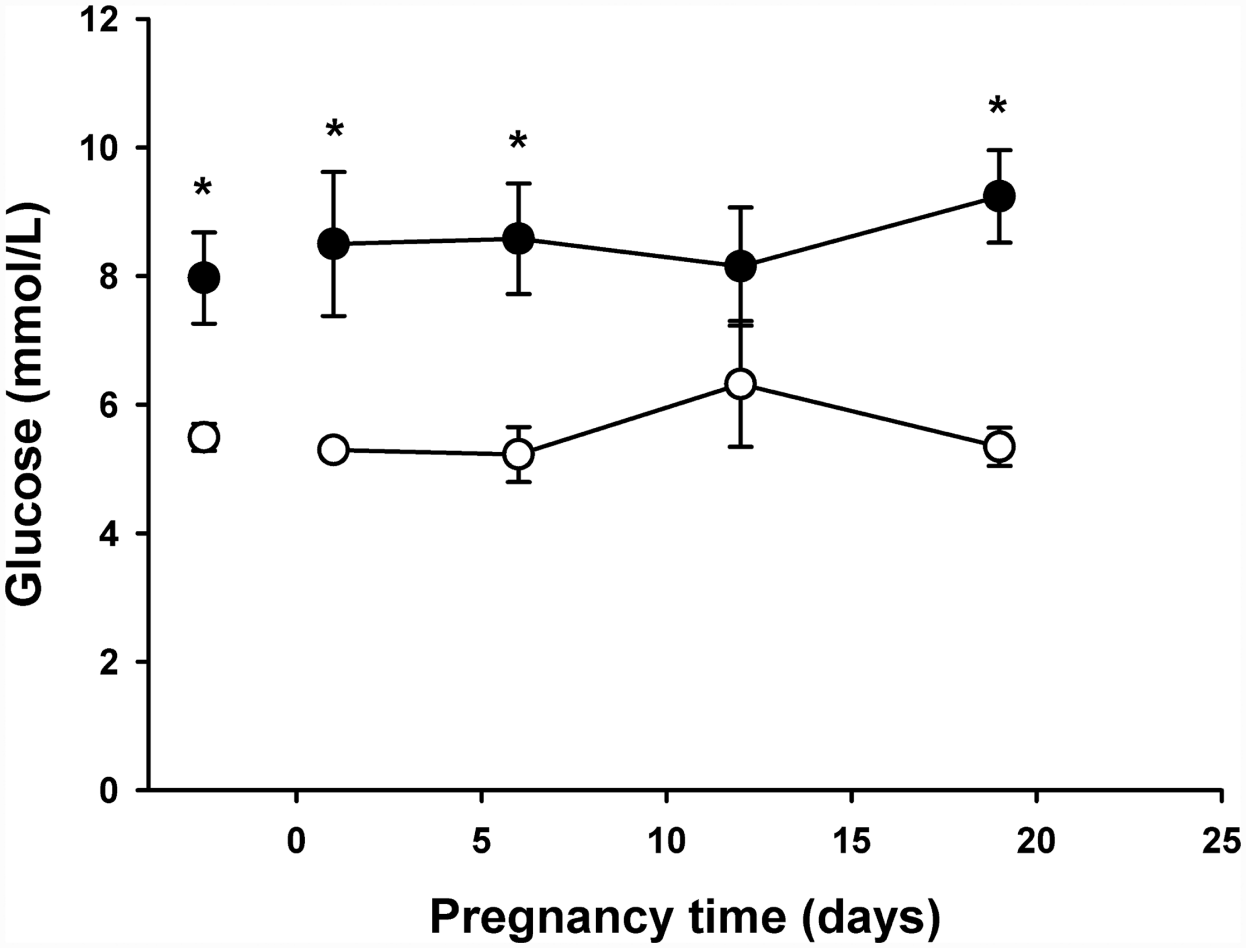
Glucose concentration during pregnancy in control (white circles) and hyperglycemic (black circles) rats. Isolated circles represent the average glycemia for the last 5 weeks previous to pregnancy. n = 10. * *p* < 0.05.

**Fig 3:**
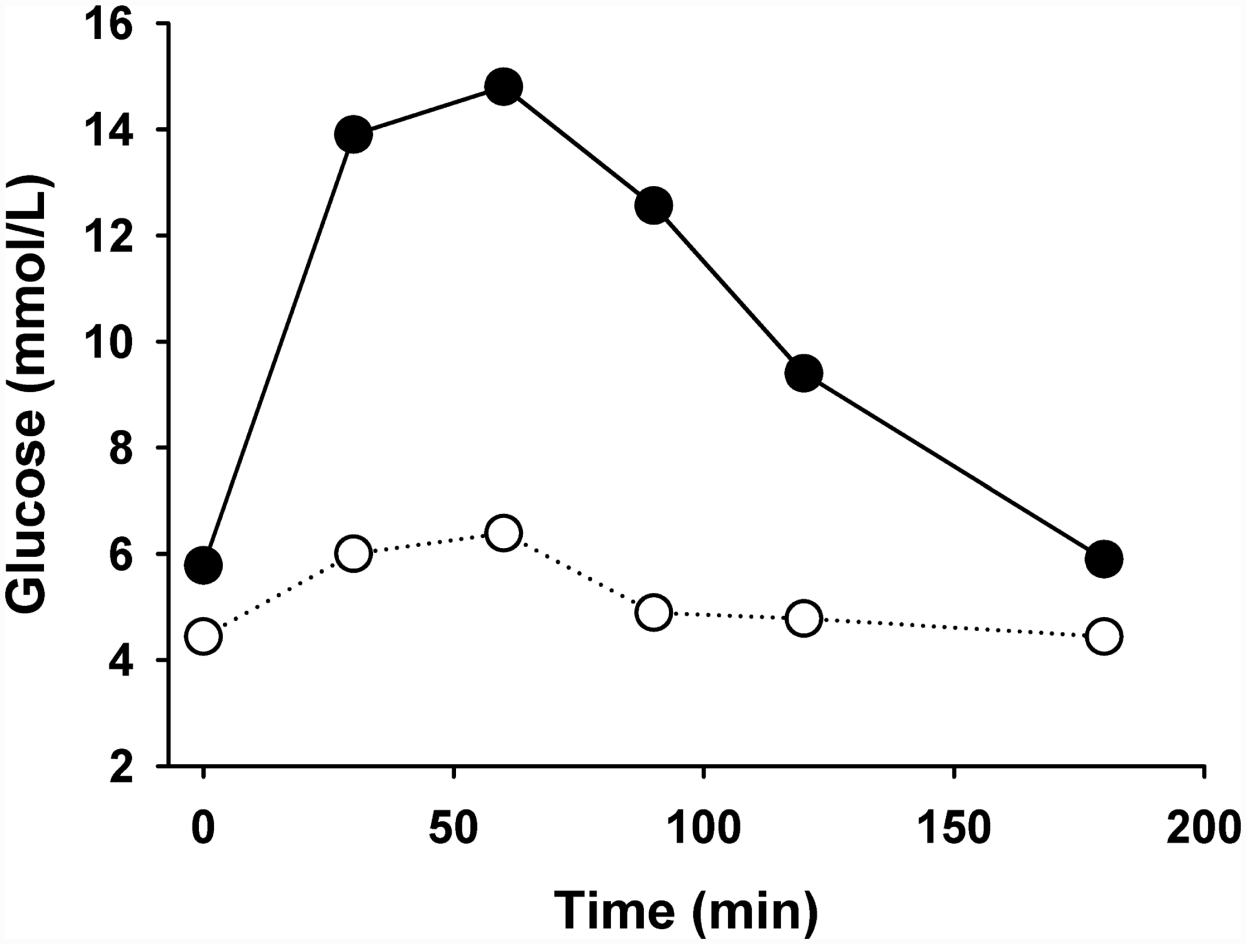
Representative glucose tolerance curves at day 18 of pregnancy of control (white circles) and hyperglycemic rats (black circles).

**Fig 4:**
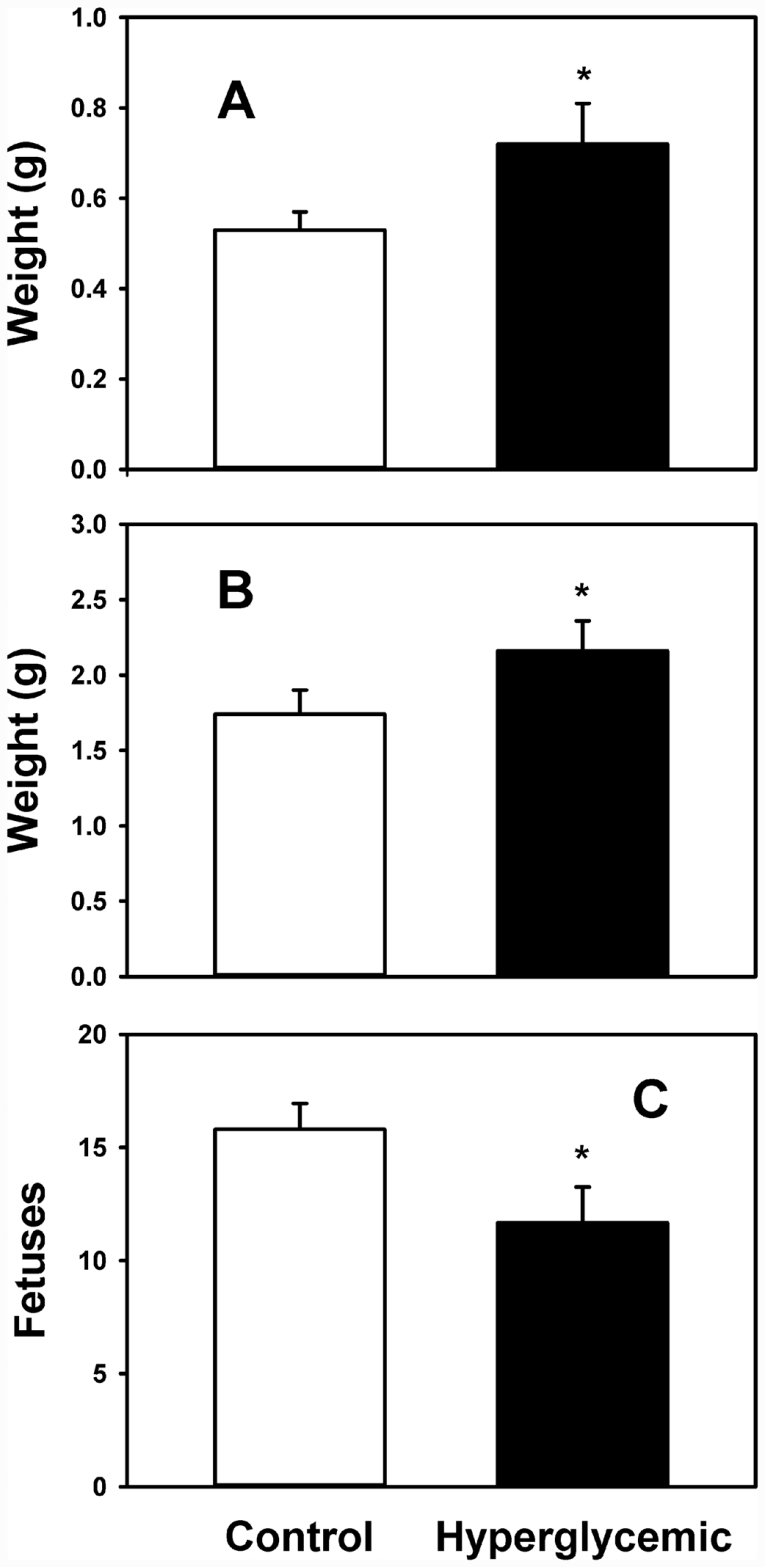
Placenta weight (A), fetuses weight (B) and number of fetuses (C) of control (white bars) and hyperglycemic rats (black bars). n = 10. *p* < 0.05.

As shown in Fig 1, female rats induced to diabetes reached sexual maturity around 16 weeks-old, which means a delay of 6–7 weeks with reference to control group. In induced rats (Fig 2) blood glucose was steady and chronically elevated previous to pregnancy, averaging 8 mmol/L in the previous month (isolated point) and 8.6 mmol/L during the gestational time (with maximal and minimal between 6.8–10.2 mmol/L), which is regarded as a mild hyperglycemia (around 155 mg/dL); however, they shown also marked glucose intolerance during pregnancy (Fig 3), it is blood glycemia concentrations around 15 mmol/L one hour after a glucose challenge with 3 g/kg body weight, orally by catheter direct to stomach, which indicates difficulty to process glucose uptake due to diabetes. Control rats had blood glycemia around 5 mmol/L in the same periods of time, and a rapid control of glycemia after the glucose challenge with 3 g/kg body weight. As shown in Fig 4, at day 19 of pregnancy the average weight of placenta and fetuses were lower for control rats, whereas the number of pups was higher.

### Lipoperoxidation

**Fig 5:**
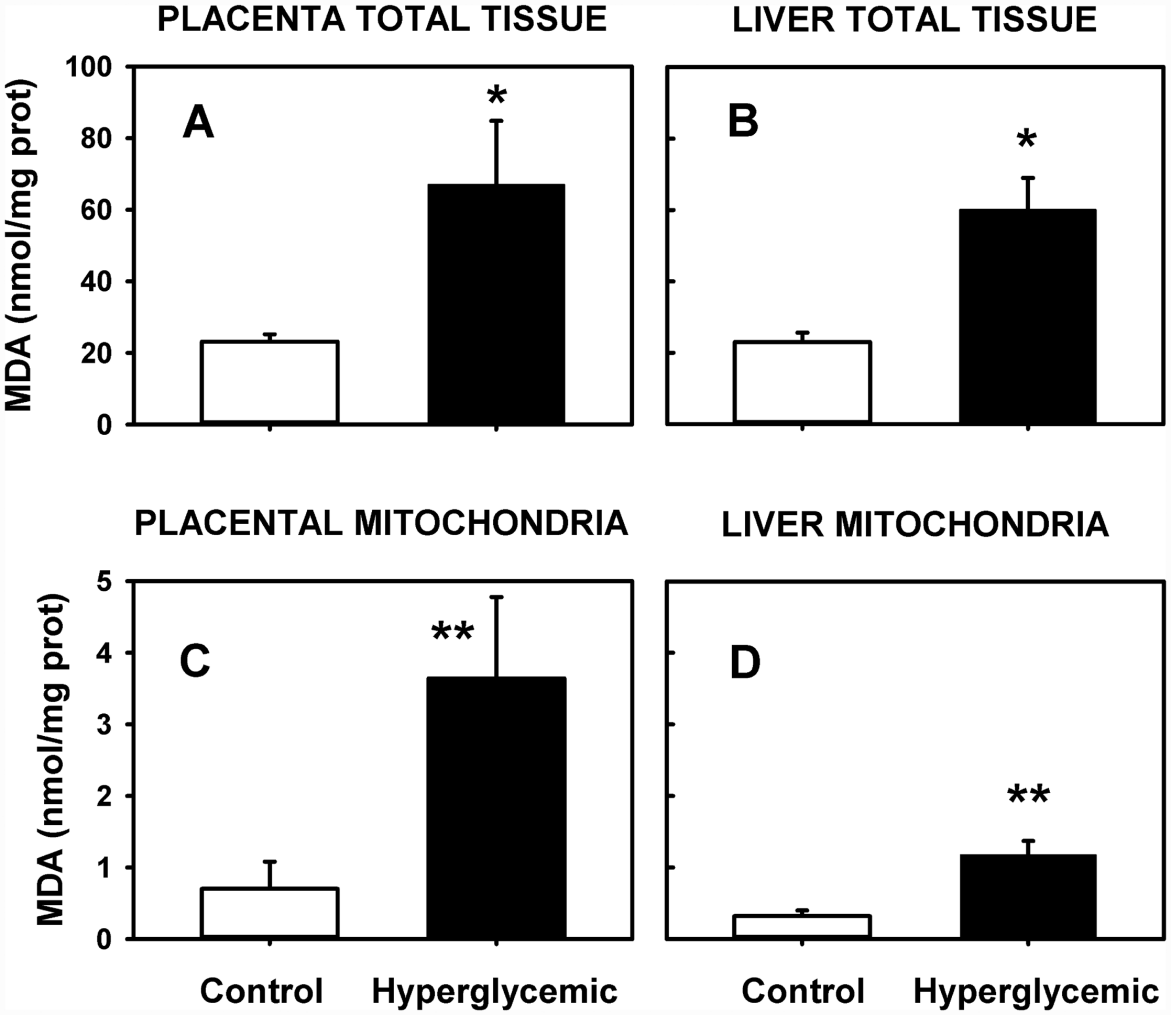
Lipoperoxidation, represented as MDA, in control (white bars) and hyperglycemic (black bars) rats. It is shown the MDA in placenta (A) and liver (B) from total tissue determinations, and mitochondria from placenta (C) and liver (D). For A and B, n = 6; *p < 0.05. For C and D, n = 5; **p <0.05.

In Fig 5A and 5B it is shown MDA content measured in placenta and liver total tissues from diabetic and control rats, whereas in lower panels (Fig 5C and 5D) it is shown the MDA in mitochondria from placenta and liver, in the same groups of rats, respectively. It is clear that this mild hyperglycemia (black bars) is enough to increase MDA production relative to control in all cases. In placenta and liver tissue there was a 3 fold MDA increase by the mild hyperglycemia, but in liver mitochondria there was a 4 fold MDA enhance and in placental mitochondria a 5 fold MDA increase. Despite the fact that the liver is a very active organ in the metabolism, lipoperoxidation in placental mitochondria was increased by the hyperglycemia.

### Mitochondrial function

In Table 1 it is shown the respiratory control ratio (RCR) of placental mitochondria from control and hyperglycemic rats. It is shown a respiratory control of 2.7±0.3 in normal rats and 1.8±0.1 in placental mitochondria from hyperglycemic rats, which means a significant reduction of 33.2% (*p < 0*.*05*) in the mitochondrial function of the hyperglycemic group. The rest of the mitochondrial parameters also point out to the same result. Hyperglycemia affects profoundly the function of placental mitochondria.

**Table 1:** Respiration of placental mitochondria from control and hyperglycemic rats.

	RCR	State 3nAO_2_/min/mgP	State 4nAO_2_/min/mgP	ADP/O	OPR
**Control**	**2.7±0.3**	**51.5±2.8**	**17.2±6.6**	**1.03±0.06**	**217.9±11.6**
**Hyperglycemic**	**1.8±0.1** *	**134.8±5.8** *	**75.4±6.9** *	**0.67±0.03** *	**166.5±7.2** *

RCR: Respiratory Control Ratio. OPR: Oxidative Phosphorilation Rate. n = 5;

* *p* < 0.05

### Fatty acids composition and mitochondrial membrane fluidity

**Fig 6:**
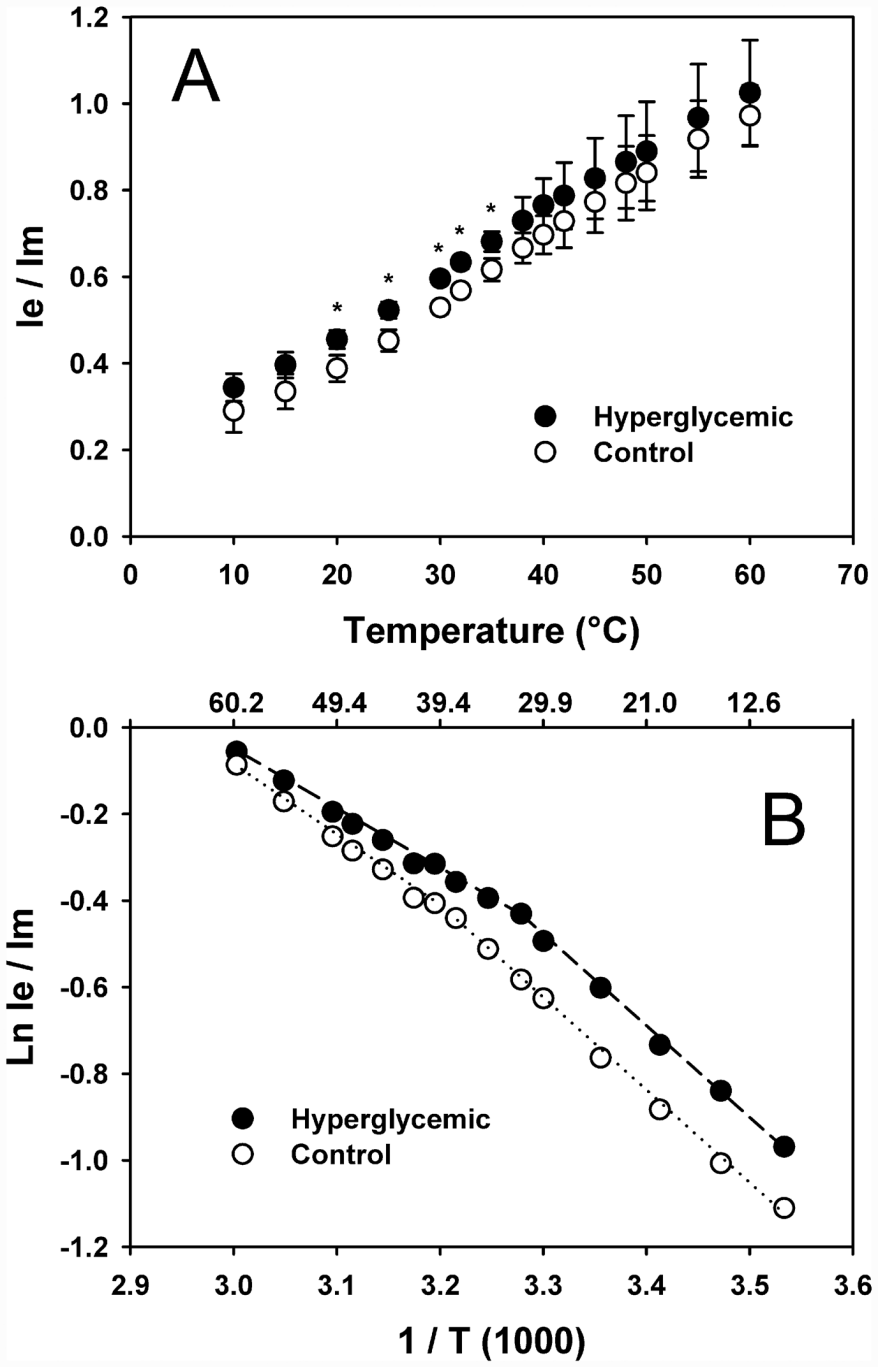
**A) Excimer to monomer (Ie/Im) fluorescence emissions of DPyP in placental mitochondria membranes from control (white circles) and hyperglycemic (black circles) rats at different temperatures. B) Arrhenius plot of individual data**. In A, for control, n = 5; for hyperglycemic, n = 6. * *p* < 0.05.

In Table 2 it is shown the fatty acids composition of total placental tissue and placental mitochondria from control and hyperglycemic rats. There were several significant changes in fatty acids composition; in placental tissue hyperglycemia increased saturated fatty acids such as palmitic, stearic, and miristic acids, and decreased some unsaturated such as linoleic, and arachidonic acids in such a way that the unsaturated to saturated ratio (U/S) index was lower in hyperglycemic animals (0.71±0.22) than in control rats (1.07±0.03), indicating a decrease in membrane fluidity in the hyperglycemic rats. Unexpectedly, in mitochondria the mild hyperglycemia moderately decreased palmitic and stearic acids, and increased the unsaturated fatty acids oleic, arachidonic, EPA, and DHA, giving a net increase in U/S to 1.20±0.04, higher than control mitochondria (1.06±0.03). These results point out to a decrease in membrane fluidity in total tissue membranes, but an increase in placental mitochondrial membranes. In order to know if fatty acids fluidity index U/S readily reflects the physical phenomenon taking place in mitochondrial membranes, we used the fluorescent probe DPyP to measure mitochondrial membrane fluidity. In Fig 6A it is shown the average excimer to monomer (Ie/Im) fluorescence emissions of DPyP at different temperatures, from 10 to 60°C, in mitochondrial membranes from control and hyperglycemic rat placentas. As Ie/Im is directly related to the membrane fluidity, it is clear that mitochondria from hyperglycemic rats were more fluid than control in average, especially at temperatures between 20 to 38°C (*p* < 0.05). At higher or lower temperatures there were not statistical differences, but still the average mitochondrial membrane fluidity in diabetic rats was greater than controls. Data from each experiment giving rise to excimerization of DPyP (Ie/Im) vs. temperature in Fig 6A was analyzed individually by the Arrhenius plot to find out the average activation energies and transition temperatures of membranes from both groups. In the Fig 6B it is shown a representative Arrhenius plot of the individual data and the average results are presented in Table 3. The Ln Ie/Im dependence of temperature, essentially exhibited a biphasic plot; the breaking points were taken as the transition temperature. The activation energies were lower in mitochondria from hyperglycemic rats (Table 3), consistent with higher membrane fluidity. The transition temperature decreased 7.8°C in mitochondria from hyperglycemic rat placenta, which is consistent with the increase in membrane fluidity.

**Table 2:** Fatty acids composition (mol%) of total lipids from placenta (tissue), and placental mitochondria, from control and hyperglycemic rats at 19 days of pregnancy.

	Tissue		Mitochondria	
Fatty acid	Control	Hyperglycemic	Control	Hyperglycemic
Miristic	0.53±0.08	0.70±0.011*	0.31±0.01	0.29±0.04
Palmitic	23.32±0.95	28.16±5.98	23.86±0.11	23.18±0.36*
Palmitoleic	1.20±0.11	1.38±0.09*	1.59±0.01	1.53±0.13
Stearic	21.15±1.60	26.68±3.43*	21.27±0.81	18.78±0.89*
Oleic	9.56±0.31	11.55±1.45*	9.02±0.01	11.76±0.44*
Linoleic	17.24±0.66	13.44±2.73*	18.42±0.22	17.01±0.44*
γ-Linoleic	ND	ND	0.13±0.00	0.09±0.00
Linolenic	ND	ND	0.15±0.00	0.26±0.04*
Arachidic	ND	ND	0.28±0.04	0.19±0.04*
Arachidonic	16.64±1.13	9.27±5.14*	16.61±0.09	17.30±0.48*
EPA	0.35±0.15	0.60±0.21	0.06±0.07	0.13±0.09
DHA	3.10±0.34	1.47±0.67*	2.36±0.01	2.82±0.13*
Unidentified	6.90±1.63	5.75±2.45	5.91±0.31	6.67±0.68
U/S	1.07±0.03	0.71±0.22*	1.06±0.03	1.20±0.04*

EPA, Eicosapentaenoic acid; DHA, Docosahexaenoic acid; U/S, unsaturated/saturated. N.D. Non Detected

n = 10;

* *p* < 0.05.

**Table 3:** Thermothropic characteristics of placental mitochondria membranes from control and hyperglycemic rats.

Group	Activation Energy(kcal/mol)		Phase Transition Temperature (°C)
	Gel	Liquid-Cristal	
Control	23.0 ± 5.0	13.9 ± 1.4	37.8 ± 1.8
Hyperglycemic	18.2 ± 4.0*	12.0 ± 0.4*	30.0 ± 2.2*

For control, n = 5; for hyperglycemic, n = 6; statistical significance:

*(*p* < 0.05).

## Discussion

In this work it was generated a model of female rats with chronic mild hyperglycemia, averaging around 8.6 mmol/L during pregnancy, and variations between 6.8 to 10.2 mmol/L, like those found in undiagnosed people as diabetics or diabetic mothers untreated. It is shown that despite being a mild hyperglycemia, there was a delay in the onset to sexual maturity by 6–7 weeks, decrease in the number of pups, but they were heavier than control, as well as the placenta from diabetic group. The glucose tolerance curve evidenced the difficulty in controlling blood glucose in the hyperglycemic group. All of those characteristics usually are not detected because of the lack of symptoms or sometimes because such glycemia is regarded as almost innocuous and close to normal, but the effects could drive the future damage to the mother and fetuses; one of those effects was the delay in reaching sexual maturity. The chronic mild hyperglycemia lasted for 16 weeks before pregnancy; afterwards, hyperglycemia continued during pregnancy. As a result of this mild gestational hyperglycemia, lipoperoxides increased 3 to 5 fold, being in placental mitochondria the highest increment. As a consequence of hyperglycemia and the resulting lipoperoxidation, the respiratory control decreased in placental mitochondria, indicating loosening of functionality. It is well known that pregnancy is a condition associated with oxidative stress resulting from the increased placental mitochondrial activity and ROS production and, in pathological conditions, such as diabetes, the stressing condition is amplified in placenta [7] as we also showed in this work for placenta and its mitochondria. The lipid peroxidation is an autooxidative process started by free radicals and the cell membrane unsaturated fatty acids are very susceptible to them. This process is characterized by unstable lipoperoxides and hydroperoxides formation, capables of reaction propagation. Associated to lipoperoxidation, several authors have found increase in rigidity of membrane phospholipids [25–27], here, the higher lipoperoxidation in placental mitochondrial membranes from diabetic animals did not decrease membrane fluidity, and contrary to expectations, at 19 days of pregnancy it shows to be more fluid than controls according to the measurements with DPyP and the fluidity index U/S (Table 2 and Fig 6). Pregnancy is a unique condition that deserves to be studied in detail in pathological conditions such as diabetes and it is possible that at earlier times, there is lower mitochondrial membrane fluidity. If that is so, the hyperfluidity we found could be a compensatory response of the mitochondria to the rigidity expected from lipoperoxides; in the same way, it could be expected that at higher hyperglycemias or longer times of exposure to hyperglycemia, the membrane fluidity should be decreased.

According to the effect of lipoperoxides on membranes, lower membrane fluidity should be measured in placental mitochondria, but it is not. In heart mitochondria, known for its high ROS production, using the fluorescent probe 1-6-diphenyl-1,3,5-hexatriene (DPH), it was reported also that mitochondrial membrane fluidity of diabetic rats was higher than control heart mitochondria; however, when 1-(4-trimethylaminophenyl)-6-phenyl-1,3,5-hexatriene (TMA-DPH) was used, there were not significant differences between the two groups, control and diabetic [11]. DPH is located preferentially at the bilayer core, whereas TMA-DPH is closer to the polar region, which means that the detected changes took place mainly at the bilayer core, but they were not important at the interphase. In this paper we used DPyP, which detects mainly the fluidity in an intermediate region between the one detected by DPH and TMA-DPH. It has been documented the decrease of membrane fluidity due to diabetes [23,28,29] but in mitochondria with high ROS production it seems that there is not such decrease in membrane fluidity. It has been recognized that DPyP is sensitive to the lipid/protein molar ratio [30] and cholesterol content in membranes [31], but cholesterol is not so abundant in mitochondria.

It results interesting to consider that due to their putative evolutionary origin from bacteria, mitochondria could be compensating the rigidifying effects of lipoperoxides on membrane fluidity increasing unsaturated fatty acids in its membranes, such as bacteria do it by low temperature [32], to cope with a lower membrane fluidity.

The relevance of changes in membrane physical properties is due to their correlation with several cell functions including the activity of cell membrane associated enzymes, solute transport, and cellular signaling induced by hormones. Both, lipid peroxidation and membrane fluidity have been implicated in diseases and aging physiology [26,33]. Here, the hyperglycemia leads to changes in thermotropic characteristics of placental mitochondrial membranes (lower activation energies and transition temperature). The increment in mitochondrial membrane fluidity might be stimulating activity of several enzymes and translocators such as the carnityl palmitoyl transferase and the citrate translocator [34], also, it might be related with the increment in mitochondrial metabolism and free radical production and/or a mild uncoupling of the electron transport chain or the reverse transport chain [35], contributing to the decreased mitochondrial respiratory control (Table 1). It has been suggested [36] that the increment in mitochondrial free radicals concentration is related with the increase in membrane fluidity. In this paper we found that there is a 5-fold increment in lipoperoxidation in mitochondria from the diabetic group (*p < 0*.*05*). In the same way, we observed that the mitochondrial respiratory index in the diabetic group increases oxygen consumption in the state 4 suggesting uncoupling in the electron transport chain.

Currently, it is recognized that placental lipid metabolism in diabetic women is altered [37,38] and the results found here confirms the same in a model of diabetes in rats. In total tissue, the U/S index is lower in hyperglycemic than control rats (0.71±0.22 vs. 1.07±0.02, respectively), showing alteration in lipid metabolism, besides of possible decrease in membrane fluidity. Recently, it was reported that there is an increase in triglycerides accumulation in human placenta by high glucose concentrations and a decrease in fatty acids oxidation [39], and it is known that desaturase activity decreases during diabetes [40]. Taking together the former information, it is possible that the U/S index decrease in hyperglycemic animals due to the accumulation of triglycerides in placenta but the esterified fatty acids must be mainly saturated. It has been stated that placenta has very low desaturase activities [41,42], in such a way that it is not possible for placenta to maintain the same U/S ratio as control rats. Besides, although fatty acids metabolism in placenta is not well understood, it is known that placenta is mainly dedicated to fatty acids transport instead of their synthesis [43].

## Conclusions

The results shown here demonstrate that even a mild hyperglycemia is capable of delaying the onset of sexual maturity driving to the typical characteristics of a pregnancy in a mother with type 2 diabetes. It is also shown a decrease in the activity of placental mitochondria accompanied of increase in lipoperoxidation and particular physical-chemical characteristics of the mitochondrial membranes making them more fluid than control, and the changes seems to be directly correlated to fatty acids composition. More work need to be done in order to find out the mechanism driving to such particular response in placental mitochondria.

## Supporting Information

S1 Fig Time for sexual maturity. (PDF)

S2 Fig Glucose concentration during pregnancy. (PDF)

S3 Fig Glucose tolerance curves. (PDF)

S4 Fig Fetus weight, placenta weight. (PDF)

S5 Fig Lipoperoxidation. (PDF)

S6 Fig Excimer to monomer fluorescence. (PDF)

S1 Table Respiration of placental mitochondria. (PDF)

S2 Table Fatty acids composition. (PDF)

S3 Table Thermotropic characteristics of mitochondrial. (PDF)
