# Supplementary material for: Even a Chronic Mild Hyperglycemia Affects Membrane Fluidity and Lipoperoxidation in Placental Mitochondria in Wistar Rats
Source: PLoS One. 2015 Dec 2;10(12):e0143778. doi: 10.1371/journal.pone.0143778 (PMC4667935; doi:10.1371/journal.pone.0143778)
Supplement: S1 Fig — (PDF) [file pone.0143778.s001.pdf]

Figure 1. Time for rats to reach sexual maturity

Data

	Control (Days)	Hyperglycemic (Days)	Control (Weeks)	Hyperglycemic (weeks)
	60	110	8.57	15.71
	65	113	9.29	16.14
	62	112	8.86	16.00
	63	109	9.00	15.57
	65	100	9.29	14.29
	67	113	9.57	16.14
	65	110	9.29	15.71
	64	110	9.14	15.71
	62	98	8.86	14.00
	66	123	9.43	17.57
Average	**63.90**	**109.80**	**9.13**	**15.69**
SD	**2.13**	**6.96**	**0.30**	**0.99**

n = 10
