# Supplementary material for: Even a Chronic Mild Hyperglycemia Affects Membrane Fluidity and Lipoperoxidation in Placental Mitochondria in Wistar Rats
Source: PLoS One. 2015 Dec 2;10(12):e0143778. doi: 10.1371/journal.pone.0143778 (PMC4667935; doi:10.1371/journal.pone.0143778)
Supplement: S2 Fig — (PDF) [file pone.0143778.s002.pdf]

Figure 2 Glucose concentrations during pregnancy.

Data

**Control**	Glycemia (mmol/L) Before pregnancy. Average of 5 weeks	Glycemia (mmol/L) during Pregnancy (days)				
		**1**	**6**	**12**	**19**	Days
	5.3	5.4	4.7	7	5.5	
	5.2	5.3	5.6	5.7	5.6	
	5.8	5.3	4.8	7.7	5.3	
	5.5	5.1	5.7	5.3	4.8	
	5.7	5.3	5.3	5.8	5.5	
	5.2	5.4	4.7	7	5.5	
	5.6	5.4	5.6	5.7	5.6	
	5.4	5.3	4.8	7.9	5.3	
	5.5	5.1	5.7	5.3	4.8	
	5.7	5.3	5.3	5.8	5.5	
Average	**5.490**	**5.29**	**5.22**	**6.32**	**5.34**	
SD	**0.213**	**0.11**	**0.43**	**0.98**	**0.30**	

**Hyperglycemic**	Glycemia (mmol/L) Before pregnancy. Average of 5 weeks	Glycemia (mmol/L) during Pregnancy (days)				
		**1**	**6**	**12**	**19**	days
	8.0	6.8	8.4	9.1	8.8	
	8.6	8.0	7.1	7.0	10.2	
	7.2	9.2	9.6	9.1	9.9	
	8.8	9.5	9.2	7.4	8.1	
	8.4	9.1	8.0	8.0	9.3	
	8.6	6.5	9.0	8.0	9.8	
	6.7	8.0	8.0	7.1	9.8	
	7.4	9.5	9.4	9.3	9.0	
	8.4	9.2	9.4	7.4	8.2	
	7.6	9.2	7.7	9.1	9.3	
Average	**7.97**	**8.50**	**8.58**	**8.15**	**9.24**	
SD	**0.71**	**1.12**	**0.86**	**0.92**	**0.72**	

n = 10
