# Supplementary material for: Even a Chronic Mild Hyperglycemia Affects Membrane Fluidity and Lipoperoxidation in Placental Mitochondria in Wistar Rats
Source: PLoS One. 2015 Dec 2;10(12):e0143778. doi: 10.1371/journal.pone.0143778 (PMC4667935; doi:10.1371/journal.pone.0143778)
Supplement: S3 Fig — (PDF) [file pone.0143778.s003.pdf]

Figure 3. Representative Glucose Tolerance Curve of control and hyperglycemic rats

Data

Glucose Tolerance Curve

Time (min)	0	30	60	90	120	180
Control	4.44	6.00	6.39	4.89	4.78	4.44
Hyperglycemic	5.78	13.90	14.80	12.53	9.40	5.90
