# Supplementary material for: Even a Chronic Mild Hyperglycemia Affects Membrane Fluidity and Lipoperoxidation in Placental Mitochondria in Wistar Rats
Source: PLoS One. 2015 Dec 2;10(12):e0143778. doi: 10.1371/journal.pone.0143778 (PMC4667935; doi:10.1371/journal.pone.0143778)
Supplement: S4 Fig — (PDF) [file pone.0143778.s004.pdf]

Figure 4. Fetus weight, placenta weight, and number of fetuses

Data

	Control			Hyperglycemic			
	**Average Fetus Weight (g)**	**Average Placenta Weight (g)**	**Number of Fetuses**		**Average Fetus Weight (g)**	**Average Placenta Weight (g)**	**Number of Fetuses**
	1.69	0.56	15		2.50	0.75	11
	1.58	0.48	15		2.14	0.79	12
	1.77	0.56	17		1.99	0.54	11
	1.92	0.61	15		1.99	0.88	13
	1.76	0.52	18		2.35	0.71	10
	1.89	0.49	14		2.46	0.81	9
	1.75	0.52	16		2.43	0.70	13
	1.99	0.51	16		2.12	0.70	11
	1.59	0.48	16		1.96	0.66	12
	1.50	0.57	16		1.99	0.68	14
**Average**	**1.74**	**0.53**	**15.80**	**Average**	**2.16**	**0.72**	**11.67**
**SD**	**0.16**	**0.04**	**1.14**	**SD**	**0.20**	**0.09**	**1.58**

n = 10
