# Supplementary material for: Even a Chronic Mild Hyperglycemia Affects Membrane Fluidity and Lipoperoxidation in Placental Mitochondria in Wistar Rats
Source: PLoS One. 2015 Dec 2;10(12):e0143778. doi: 10.1371/journal.pone.0143778 (PMC4667935; doi:10.1371/journal.pone.0143778)
Supplement: S5 Fig — (PDF) [file pone.0143778.s005.pdf]

Figure 5 Lipoperoxidation, represented as MDA

Data

	**Placenta Total Tissue** MDA (nmol/mg prot)		**Liver Total Tissue** MDA (nmol/mg prot)	
	Control	Hyperglycemic	Control	Hyperglycemic
	25.6	88.8	24.8	51.2
	24.0	73.6	26.4	58.4
	20.0	64.0	23.0	72.8
	24.8	40.0	20.0	49.6
	21.6	80.8	20.0	66.0
	23.0	55.2	24.0	62.4
Average	**23.17**	**67.07**	**23.03**	**60.07**
SD	**2.09**	**17.81**	**2.60**	**8.87**

n = 6

	**Placental mitochondria** MDA (nmol/mg prot)		Liver mitochondria MDA (nmol/mg prot)	
	Control	Hyperglycemic	Control	Hyperglycemic
	1.2	3.8	0.3	1.4
	0.5	4.7	0.4	1.3
	0.3	2.1	0.4	1.1
	1.0	2.9	0.3	1.2
	0.5	4.7	0.2	0.9
Average	**0.70**	**3.64**	**0.32**	**1.18**
SD	**0.38**	**1.14**	**0.08**	**0.19**

n = 5
