# Supplementary material for: Even a Chronic Mild Hyperglycemia Affects Membrane Fluidity and Lipoperoxidation in Placental Mitochondria in Wistar Rats
Source: PLoS One. 2015 Dec 2;10(12):e0143778. doi: 10.1371/journal.pone.0143778 (PMC4667935; doi:10.1371/journal.pone.0143778)
Supplement: S6 Fig — (PDF) [file pone.0143778.s006.pdf]

Figure 6. Excimer to monomer (Ie/Im) fluorescence emissions of DPyP in placental mitochondria

Data

Control

**Temp (°C)**	**Ie / Im**					**Average**	**SD**
10	0.286	0.330	0.329	0.272	0.229	**0.289**	**0.043**
15	0.354	0.365	0.365	0.329	0.280	**0.339**	**0.036**
20	0.412	0.409	0.414	0.384	0.346	**0.393**	**0.029**
25	0.481	0.469	0.466	0.460	0.415	**0.458**	**0.025**
30	0.565	0.523	0.535	0.547	0.512	**0.536**	**0.021**
32	0.614	0.564	0.558	0.585	0.565	**0.577**	**0.023**
35	0.644	0.591	0.599	0.647	0.628	**0.622**	**0.026**
38	0.709	0.630	0.644	0.697	0.696	**0.675**	**0.036**
40	0.714	0.653	0.666	0.725	0.743	**0.700**	**0.039**
42	0.761	0.677	0.675	0.769	0.792	**0.735**	**0.055**
45	0.784	0.710	0.720	0.803	0.859	**0.775**	**0.062**
48	0.832	0.743	0.752	0.848	0.922	**0.819**	**0.074**
50	0.861	0.773	0.777	0.857	0.955	**0.845**	**0.075**
55	0.938	0.845	0.843	0.964	1.021	**0.922**	**0.077**
60	0.983	0.910	0.917	1.005	1.056	**0.974**	**0.061**

n = 5

Hyperglycemic

**Temp (°C)**	**Ie / Im**						**Average**	**SD**
10	0.276	0.443	0.363	0.379	0.316	0.317	**0.349**	**0.059**
15	0.334	0.485	0.410	0.432	0.371	0.372	**0.401**	**0.053**
20	0.396	0.529	0.465	0.480	0.437	0.439	**0.458**	**0.045**
25	0.465	0.576	0.526	0.548	0.504	0.514	**0.522**	**0.038**

30	0.542	0.618	0.588	0.610	0.585	0.600	**0.590**	**0.027**
32	0.584	0.647	0.620	0.650	0.621	0.643	**0.627**	**0.025**
35	0.615	0.666	0.666	0.674	0.670	0.715	**0.668**	**0.032**
38	0.666	0.711	0.684	0.700	0.727	0.807	**0.716**	**0.050**
40	0.687	0.726	0.713	0.730	0.767	0.851	**0.746**	**0.058**
42	0.719	0.745	0.726	0.730	0.804	0.889	**0.769**	**0.066**
45	0.748	0.775	0.746	0.771	0.838	0.955	**0.805**	**0.080**
48	0.807	0.815	0.776	0.800	0.871	1.013	**0.847**	**0.087**
50	0.827	0.839	0.801	0.823	0.881	1.054	**0.871**	**0.094**
55	0.904	0.898	0.873	0.884	0.970	1.141	**0.945**	**0.102**
60	0.938	0.958	0.931	0.945	1.030	1.195	**0.999**	**0.102**

n = 6

From these information, it was performed the Arrhenius analysis to each group of data for obtaining the thermotropic characteristics of mitochondrial membranes i.e. activation energies and phase transition temperatures.

En Fig 6B it is presented a representative Arrhenius plot.

Data for the Arrhenius plot in Figure 6B

Control

**Temp (°C)**	**Ie/Im**	**Temp (°K)**	**1/T (1000)**	**Ln Ie/Im**
10	0.329	283.15	**3.5317**	**-1.1103**
15	0.365	288.15	**3.4704**	**-1.0070**
20	0.414	293.15	**3.4112**	**-0.8829**
25	0.466	298.15	**3.3540**	**-0.7631**
30	0.535	303.15	**3.2987**	**-0.6261**
32	0.558	305.15	**3.2771**	**-0.5826**
35	0.599	308.15	**3.2452**	**-0.5119**
38	0.644	311.15	**3.2139**	**-0.4407**
40	0.666	313.15	**3.1934**	**-0.4063**
42	0.675	315.15	**3.1731**	**-0.3936**

45	0.720	318.15	**3.1432**	**-0.3285**
48	0.752	321.15	**3.1138**	**-0.2851**
50	0.777	323.15	**3.0945**	**-0.2518**
55	0.843	328.15	**3.0474**	**-0.1713**
60	0.917	333.15	**3.0017**	**-0.0867**

Hyperglycemic

Temp (°C)	Ie/Im	Temp (°K)	1/T (1000)	Ln Ie/Im
10	0.379	283.15	**3.5317**	**-0.9690**
15	0.432	288.15	**3.4704**	**-0.8397**
20	0.480	293.15	**3.4112**	**-0.7336**
25	0.548	298.15	**3.3540**	**-0.6017**
30	0.610	303.15	**3.2987**	**-0.4938**
32	0.650	305.15	**3.2771**	**-0.4308**
35	0.674	308.15	**3.2452**	**-0.3944**
38	0.700	311.15	**3.2139**	**-0.3573**
40	0.730	313.15	**3.1934**	**-0.3153**
42	0.730	315.15	**3.1731**	**-0.3144**
45	0.771	318.15	**3.1432**	**-0.2602**
48	0.800	321.15	**3.1138**	**-0.2232**
50	0.823	323.15	**3.0945**	**-0.1953**
55	0.884	328.15	**3.0474**	**-0.1228**
60	0.945	333.15	**3.0017**	**-0.0564**
