# Supplementary material for: Even a Chronic Mild Hyperglycemia Affects Membrane Fluidity and Lipoperoxidation in Placental Mitochondria in Wistar Rats
Source: PLoS One. 2015 Dec 2;10(12):e0143778. doi: 10.1371/journal.pone.0143778 (PMC4667935; doi:10.1371/journal.pone.0143778)
Supplement: S1 Table — (PDF) [file pone.0143778.s007.pdf]

<u>Table 1.</u> Placenta Mitochondrial activity in control and hyperglycemic rats

Data

				**nA°O2/min/mg P**					**ADP:O**			**OPR**	
	**Respiratory Control Ratio**			**State 3**		**State 4**						ADP nmol/mg [P]/ min	
	Control	Hyperglyc		Control	Hyperglyc	Control	Hyperglyc		Control	Hyperglyc		Control	Hyperglyc
	2.60	1.70		48.53	136.27	18.67	80.27		0.97	0.68		230.8	164.4
	3.00	1.86		50.40	128.80	16.80	69.07		1.01	0.64		222.2	173.9
	2.33	1.86		52.27	138.13	22.40	74.67		1.05	0.69		214.3	162.2
	3.00	1.70		56.00	141.87	18.67	84.00		1.12	0.71		200.0	157.9
	2.47	1.86		50.40	128.80	20.53	69.07		1.01	0.64		222.2	173.9
Average	**2.68**	**1.79**		**51.52**	**134.77**	**19.41**	**75.41**		**1.03**	**0.67**		**217.90**	**166.45**
SD	0.31	0.09		2.83	5.81	2.13	6.68		0.06	0.03		11.58	7.20

n = 5
