# Supplementary material for: Even a Chronic Mild Hyperglycemia Affects Membrane Fluidity and Lipoperoxidation in Placental Mitochondria in Wistar Rats
Source: PLoS One. 2015 Dec 2;10(12):e0143778. doi: 10.1371/journal.pone.0143778 (PMC4667935; doi:10.1371/journal.pone.0143778)
Supplement: S2 Table — (PDF) [file pone.0143778.s008.pdf]

Table 2. Fatty acid composition (mol%) of total lipids from placenta (total tissue) and placental mitochondria.

Data

Placenta Total Tissue

Control	mol %										Average	SD
Miristic	0.60	0.50	0.60	0.40	0.60	0.50	0.50	0.60	0.40	0.60	**0.53**	**0.08**
Palmitic	24.60	23.20	24.60	22.60	22.30	23.20	23.20	24.60	22.60	22.30	**23.32**	**0.95**
Palmitoleic	1.30	1.10	1.30	1.10	1.30	1.10	1.10	1.30	1.10	1.30	**1.20**	**0.11**
Stearic	19.40	21.70	19.40	20.50	23.60	21.70	21.70	19.40	20.50	23.60	**21.15**	**1.60**
Oleic	10.00	9.40	10.00	9.30	9.40	9.40	9.40	10.00	9.30	9.40	**9.56**	**0.31**
Linoleic	16.70	18.10	16.70	17.40	16.60	18.10	18.10	16.70	17.40	16.60	**17.24**	**0.66**
Arachidonic	15.10	17.10	15.10	16.90	18.00	17.10	17.10	15.10	16.90	18.00	**16.64**	**1.13**
EPA	0.20	0.30	0.20	0.40	0.60	0.30	0.30	0.20	0.40	0.60	**0.35**	**0.15**
DHA	3.20	3.00	3.20	3.60	2.60	3.00	3.00	3.20	3.60	2.60	**3.10**	**0.34**
Unidentified	8.70	5.50	8.70	8.00	5.20	5.50	5.50	8.70	8.00	5.20	**6.90**	**1.63**
Saturated (S)	44.60	45.40	44.60	43.50	46.50	45.40	45.40	44.60	43.50	46.50	**45.00**	**1.05**
Unsaturated (U)	46.50	49.00	46.50	48.70	48.50	49.00	49.00	46.50	48.70	48.50	**48.09**	**1.11**
U/S	1.04	1.08	1.04	1.12	1.04	1.08	1.08	1.04	1.12	1.04	**1.07**	**0.03**

Hyperglycemic					mol %						Average	SD
Miristic	0.80	0.60	0.70	0.70	0.80	0.60	0.70	0.90	0.60	0.60	**0.70**	**0.11**
Palmitic	29.80	29.90	20.40	35.80	19.80	29.90	20.40	35.80	29.90	29.90	**28.16**	**5.98**
Palmitoleic	1.40	1.30	1.40	1.40	1.40	1.30	1.40	1.60	1.30	1.30	**1.38**	**0.09**
Stearic	23.40	26.10	22.20	31.40	30.80	26.10	23.20	31.40	26.10	26.10	**26.68**	**3.43**
Oleic	9.80	12.50	9.30	12.30	12.50	12.50	9.30	12.30	12.50	12.50	**11.55**	**1.45**
Linoleic	15.70	14.90	15.30	9.50	9.50	14.90	15.30	9.50	14.90	14.90	**13.44**	**2.73**
Arachidonic	16.10	7.80	16.60	4.30	4.80	7.50	16.60	4.00	7.50	7.50	**9.27**	**5.14**
EPA	0.90	0.60	0.80	0.40	0.60	0.40	0.90	0.40	0.60	0.40	**0.60**	**0.21**
DHA	2.20	1.40	2.40	0.70	0.70	1.40	2.40	0.70	1.40	1.40	**1.47**	**0.67**
Unidentified	2.50	5.10	9.20	3.70	9.00	5.40	8.80	3.20	5.20	5.40	**5.75**	**2.45**
Saturated (S)	54.00	56.60	43.30	67.90	51.40	56.60	44.30	68.10	56.60	56.60	**55.54**	**8.24**
Unsaturated (U)	46.10	38.50	45.80	28.60	29.50	38.00	45.90	28.50	38.20	38.00	**37.71**	**6.99**
U/S	0.85	0.68	1.06	0.42	0.57	0.67	1.04	0.42	0.67	0.67	**0.71**	**0.22**

n = 10

Mitochondria from placenta

Control	mol %									Average	SD
Miristic	0.33	0.31	0.31	0.31	0.33	0.31	0.31	0.31	0.33	0.31	**0.31**	**0.01**
Palmitic	24.01	23.79	23.79	23.79	24.01	23.79	23.79	23.79	24.01	23.79	**23.86**	**0.11**
Palmitoleic	1.61	1.59	1.59	1.59	1.61	1.59	1.59	1.59	1.61	1.59	**1.59**	**0.01**
Stearic	20.10	21.77	21.77	21.77	20.10	21.77	21.77	21.77	20.10	21.77	**21.27**	**0.81**
Oleic	9.00	9.03	9.03	9.03	9.00	9.03	9.03	9.03	9.00	9.03	**9.02**	**0.01**
Linoleic	18.74	18.28	18.28	18.28	18.74	18.28	18.28	18.28	18.74	18.28	**18.42**	**0.22**
γ-linoleic	0.13	0.13	0.13	0.13	0.13	0.13	0.13	0.13	0.13	0.13	**0.13**	**0.00**
Linolenic	0.14	0.15	0.15	0.15	0.14	0.15	0.15	0.15	0.14	0.15	**0.14**	**0.00**
Arachidic	0.22	0.31	0.31	0.31	0.22	0.31	0.31	0.31	0.22	0.31	**0.28**	**0.04**
Arachidonic	16.73	16.55	16.55	16.55	16.73	16.55	16.55	16.55	16.73	16.55	**16.61**	**0.09**
EPA	0.15	0.02	0.02	0.02	0.15	0.02	0.02	0.02	0.15	0.02	**0.06**	**0.07**
DHA	2.34	2.36	2.36	2.36	2.34	2.36	2.36	2.36	2.34	2.36	**2.36**	**0.01**
Unidentified	6.36	5.72	5.72	5.72	6.36	5.72	5.72	5.72	6.36	5.72	**5.91**	**0.31**
Saturated (S)	44.66	46.18	46.18	46.18	44.66	46.18	46.18	46.18	44.66	46.18	**45.72**	**0.73**
Unsaturated (U)	48.84	48.10	48.10	48.10	48.84	48.10	48.10	48.10	48.84	48.10	**48.32**	**0.36**
U/S	1.09	1.04	1.04	1.04	1.09	1.04	1.04	1.04	1.09	1.04	**1.06**	**0.03**

**Hyperglycemic**					**mol %**						**Average**	**SD**
Miristic	0.28	0.34	0.25	0.28	0.25	0.28	0.34	0.28	0.25	0.34	**0.29**	**0.04**
Palmitic	23.27	23.55	22.69	23.27	22.69	23.27	23.55	23.27	22.69	23.55	**23.18**	**0.36**
Palmitoleic	1.38	1.64	1.62	1.38	1.62	1.38	1.64	1.38	1.62	1.64	**1.53**	**0.13**
Stearic	19.74	18.64	17.75	19.74	17.75	19.74	18.34	19.74	17.75	18.64	**18.78**	**0.89**
Oleic	11.13	12.02	12.08	11.13	12.08	11.93	12.02	11.13	12.08	12.02	**11.76**	**0.44**
Linoleic	16.50	17.35	17.36	16.50	17.36	16.50	17.35	16.50	17.36	17.35	**17.01**	**0.44**
γ-linoleic	0.10	0.09	0.09	0.10	0.09	0.10	0.09	0.10	0.09	0.09	**0.09**	**0.00**
Linolenic	0.31	0.23	0.24	0.31	0.24	0.31	0.23	0.31	0.24	0.23	**0.26**	**0.04**
Arachidic	0.10	0.21	0.22	0.10	0.22	0.10	0.21	0.30	0.22	0.21	**0.19**	**0.07**
Arachidonic	17.78	16.67	17.29	17.78	17.29	17.78	16.67	17.78	17.29	16.67	**17.30**	**0.48**
EPA	0.21	0.01	0.16	0.21	0.16	0.21	0.01	0.21	0.16	0.01	**0.13**	**0.09**
DHA	2.95	2.66	2.86	2.95	2.86	2.95	2.66	2.95	2.86	2.66	**2.84**	**0.13**
Unidentified	6.03	6.60	7.39	6.03	7.89	6.03	6.60	6.03	7.39	6.70	**6.67**	**0.68**
Saturated (S)	43.38	42.74	40.91	43.38	40.91	43.38	42.44	43.58	40.91	42.74	**42.44**	**1.11**
Unsaturated (U)	50.36	50.66	51.70	50.36	51.70	51.16	50.66	50.36	51.70	50.66	**50.93**	**0.58**
U/S	1.16	1.19	1.26	1.16	1.26	1.18	1.19	1.16	1.26	1.19	**1.20**	**0.04**

n = 10
