# Supplementary material for: Even a Chronic Mild Hyperglycemia Affects Membrane Fluidity and Lipoperoxidation in Placental Mitochondria in Wistar Rats
Source: PLoS One. 2015 Dec 2;10(12):e0143778. doi: 10.1371/journal.pone.0143778 (PMC4667935; doi:10.1371/journal.pone.0143778)
Supplement: S3 Table — (PDF) [file pone.0143778.s009.pdf]

Table 3. Thermotropic characteristics of placental mitochondria membranes of control and hyperglycemic rats.

Data

	Activation Energy (kJ/mol)				Phase Transition (°C)	
	Gel		Liquid-Cristal			
	**Control**	**Hyperglycemic**	**Control**	**Hyperglycemic**	**Control**	**Hyperglycemic**
	19.8	21.3	14.4	12.1	38.0	30.0
	18.6	12.3	11.5	12.1	38.0	26.0
	20.3	14.5	14.2	11.7	35.0	32.0
	25.9	18.8	14.4	11.3	38.0	30.0
	30.5	20.0	15.1	12.3	40.0	30.0
		22.6		12.2		32.0
Average	**23.0**	**18.2**	**13.9**	**12.0**	**37.8**	**30.0**
SD	**5.0**	**4.0**	**1.4**	**0.4**	**1.8**	**2.2**

Control n = 5

Hyperglycemic n = 6
